# The impact of HIV/AIDS on compliance with antidepressant treatment in major depressive disorder: A prospective study in a South African private healthcare cohort

**DOI:** 10.1186/s12981-015-0050-2

**Published:** 2015-04-14

**Authors:** Francois N Slabbert, Brian H Harvey, Christiaan B Brink, Martie S Lubbe

**Affiliations:** Medicines Usage Group (MUSA), North-West University, Potchefstroom, 2520 South Africa; Division of Pharmacology, North-West University, Potchefstroom, 2520 South Africa; Centre of Excellence for Pharmaceutical Sciences, School of Pharmacy, North-West University, Potchefstroom, 2520 South Africa

**Keywords:** Major depressive disorder, HIV/AIDS, Compliance, Antidepressants, Venlafaxine

## Abstract

**Background:**

MDD and HIV/AIDS have a high prevalence worldwide with severe consequences for patients. In both conditions, compliance with treatment is key to successfully treat these disorders. In the current study, we examine the effect of MDD on the compliance with ADs in patients diagnosed with co-morbid HIV/AIDS and how different classes of ADs influence compliance in this group of patients.

**Methods:**

A prospective, cohort study design was used to analyse nationally representative medicine claims data submitted to a privately-owned South African Pharmaceutical Benefit Management (PBM) company. Two groups were distinguished in the database, namely patients with only MDD and patients with both MDD and HIV/AIDS, over a six-year study period. The study population was determined by the following inclusion criteria: patients older than 18 years, MDD should be diagnosed by a psychiatrist supported by an appropriate ICD-10 code, and all patients have to be on combination antiretroviral treatment (cARV) treatment. The medicine possession ratio (MPR) was used as proxy to determine patient compliance with AD medication.

**Results:**

127 patients (i.e. 0.24%) met the criteria of co-morbid MDD and HIV/AIDS. Females have a significantly higher prevalence of MDD and HIV/AIDS when compared to males. Patients diagnosed with both HIV/AIDS and MDD (74.43. ± 32.03, 95% Cl: 71.51-77.34) have a statistical significantly (*p* < 0.0001) lower compliance with AD treatment vs. MDD patients (80.94% ± 29.44, 95% Cl: 80.56-81.33), but the practical significance thereof, is low (Cohen’s d = 0.2255). In this group only 26.83% of TCA had acceptable compliance compared to the 58.57% of SNRIs. Noteworthy observations were that 75% (*p* < 0.0217; Cramer’s V = 0.0388) of venlafaxine and 28.6% (*p* < 0.0197; Cramer’s V = −0.0705) of the paroxetine items were compliant in patients diagnosed with both HIV/AIDS and MDD.

**Conclusions:**

AD compliance is statistical significantly lower in depressed HIV/AIDS vs. depressed non-HIV/AIDS patients. However, these differences is of low practical or clinical significance, meaning that depressed HIV/AIDS patients would have missed approximately two AD doses (6.5% of a 30-day treatment period) more than the non-HIV/AIDS depressed patient over the same treatment period*.*

**Electronic supplementary material:**

The online version of this article (doi:10.1186/s12981-015-0050-2) contains supplementary material, which is available to authorized users.

## Background

In 2012, the UNAIDS World AIDS Day Report estimated that approximately 34 million people worldwide are infected with human immunodeficiency virus (HIV), whereas 69% of these infected patients live in sub-Saharan Africa [1]. In the United States, more than one million individuals are infected with HIV and acquired immunodeficiency syndrome (AIDS, collectively HIV/AIDS), while nearly 50 000 more contract HIV each year [2]. In South Africa, the prevalence of HIV/AIDS is estimated to be 17.3% in the age group 15 to 49 years and approximately 5.6 million people in the country are living with HIV/AIDS [3]. South Africa is one of the countries with the highest HIV/AIDS-related mortality rates in the world, with approximately 270 000 deaths reported in 2011 [3].

Apart from the huge impact on the health system, HIV/AIDS also affects individual patients, causing a great deal of suffering in both their social lives as well as mental and physical health. It is therefore not surprising that HIV/AIDS is co-morbid with various psychosocial disorders, such as major depressive disorder (MDD) [4]. MDD is one of the main causes of psychiatric morbidity in HIV/AIDS patients [4]. In a six-month follow-up study in HIV/AIDS-infected patients in South Africa, Olley and co-workers described a 34.9% prevalence of MDD [5]. Elsewhere in the literature, however, there is some controversy over the prevalence of MDD in HIV/AIDS patients, with the prevalence rate varying between 3 and 54% [4,6,7]. Further studies in this regard are therefore urgently needed.

The co-morbidity between chronic illnesses and MDD is a well-recognised phenomenon. Chronically ill patients display two to three times higher rates of MDD when compared to healthy individuals of the same age and gender [8-10]. Diabetes, asthma, epilepsy, cancer, coronary heart disease, hypertension and HIV/AIDS are among the most common chronic illnesses that are associated with an increased prevalence of MDD [11,12]. Considering the symptomatology of depression, depressed patients tend to neglect themselves due to fatigue, low energy levels, impaired memory and the sense of helplessness [13]. Patients with a chronic illness and co-morbid MDD have a significantly decreased compliance with self-care treatment regimens [14], which negatively affects long-term treatment outcomes and is ultimately associated with impaired daily functioning and a decreased quality of life [15,16].

HIV-positive patients have twice the incidence of MDD compared to HIV-negative individuals [17-20]. In particular, late stage HIV (CD4 count of <200 cells/μL) is associated with a significantly higher incidence of MDD, with a number of studies showing as much as a two and a half-fold increase in the incidence of MDD [21-24]. A recent study by Lopes and co-workers found that HIV-positive men were more likely to have a major depressive disorder/dysthymia (OR = 3.77; 95% CI, 1.16-12.27) when compared to HIV-negative males [25]. The reason for this is likely to be multifactorial, although chronic-psychosocial stress (e.g. discrimination, isolation, violence, stigmatisation, hopelessness and drug abuse) [26] associated with HIV/AIDS can induce inflammation in the central nervous system (CNS), which is a major etiological factor [27]. Exposure to chronic psychosocial stress induces the continuous release of cortisol, and activates the hypothalamic-pituitary-adrenal (HPA) axis as well as the sympathetic autonomic nervous system, which in turn activates immune cells in both CNS and in the periphery [28,29]. Depressed patients express elevated levels and an imbalance of cytokines, especially interleukin-1 (IL-1) and tumour necrosis factor alpha (TNF α), which leads to cognitive and mood impairment [30-32]. Further evidence supporting inflammation as an etiological factor in depression, is that patients receiving interferon Alfa (IFN-α) therapy for various types of cancer develop severe depression, INF being an inflammatory mediator [33-35]. Cerebral HIV infection will induce chronic inflammation in the brain and this causes the release of IFN and other cytokines, with depression as a result [27]. Therefore, HIV/AIDS patients with chronic increased cytokine levels as a result of viral infection, can be expected to be particularly vulnerable to develop depression [36].

The introduction of combination antiretroviral therapy (cART) has been successful in improving morbidity and decreasing the mortality rate of HIV/AIDS, which provides a glimmer of hope [37-39]. Importantly, however, cART can suppress HIV-1 RNA levels and increase CD4 T cell lymphocytes only if the cART is associated with excellent compliance and persistence [40-43]. Patients who are HIV positive must maintain a compliance rate of at least 95% in order to prevent virologic failure, increase CD4 T cell lymphocyte count, and decrease viral load and opportunistic infections [44-47]. Another risk associated with non-compliance with ART is the increased risk of viral resistance that ultimately leads to treatment failure [48-51]. On the other hand, compliance with AD treatment is just as important. Patients who fail to comply with their AD treatment have a twofold increased risk for relapse [52], are more likely to experience antidepressant withdrawal syndrome [53], and have a significantly increased mortality and morbidity rate associated with MDD [54]. Consequently, poor compliance with either ART or AD treatment will significantly compromise a successful treatment outcome.

The current literature regarding the prevalence of HIV/AIDS and MDD is quite limited. Therefore, this study will firstly strive to determine the prevalence of HIV/AIDS-positive patients within the MDD-diagnosed population in the private health sector of South Africa. Secondly, the study will investigate how AD compliance is affected by MDD in HIV/AIDS-positive patients when compared to depressed non-HIV/AIDS patients and, lastly, whether AD compliance has any association with gender and antidepressant class in this population.

## Results

The research cohort consisted of two groups, namely MDD patients with HIV/AIDS (n = 127) and patients with only MDD (n = 12 270) (Table 1). These patients received over the study period respectively 466 and 22 831 AD items (Table 2).

**Table 1:** **Patient demographics**

**Total number of patients in the study population**						
**Illness**	**n**	**%**				
MDD + HIV	127	0.24				
MDD	12270	22.94				
HIV	41086	76.82				
**Gender classification of the study population**						
	**Male**		**Female**			
**Illness**	**n**	**%**	**n**	**%**		
MDD + HIV	37	29.13	90	70.87		
MDD	3496	28.49	8774	71.51		
HIV	18268	44.46	22818	55.54		
**Age classification of the study population**						
	**>18 ≤** **40 years**		**>40 ≤ 60 years**		**>60 years**	
**Illness**	**n**	**%**	**n**	**%**	**n**	**%**
MDD + HIV	31	24.41	75	59.06	21	16.54
MDD	2662	21.7	21176	47.03	3837	31.27
HIV	16586	40.37	21176	5154	1652	4.02

**Table 2:** **The effect of illness, gender and age on medicine possession ratio (MPR)**

**Mean MPR of ADs in MDD patients with/without HIV/AIDS (dispensed items)**					
**Illness**	**n**	**Mean ± SD (%)**	**95% Cl**	p*	**Cohen’s d-value**
HIV/AIDS + MDD	466	74.43 ± 32.03	71.51-77.34	0.0001	0.2255
MDD	22831	80.94 ± 29.44	80.56-81.33		
**Mean MPR of ADs in MDD patients with HIV/AIDS vs. gender (dispensed items)**					
**Gender**	**n**	**Mean ± SD (%)**	**95% Cl**	p*	**Cohen’s d-value**
Female	310	69.35 ± 29.69	66.03-72.67	0.0001	0.4755
Male	156	84.51 ± 34.18	79.10-89.91		
**Mean MPR of ADs in MDD patients without HIV/AIDS vs. gender (dispensed items)**					
**Gender**	**n**	**Mean ± SD (%)**	**95% Cl**	p*	
Female	16600	80.97 ± 29.72	80.52-81.43	0.8067	
Male	6231	80.87 ± 28.65	80.16-81.58		
**Mean MPR of ADs in MDD patients with HIV/AIDS vs. age group (dispensed items)**					
**Age (years)**	**n**	**Mean ± SD (%)**	**95% Cl**	p**	
> 18 ≤ 40	126	74.57 ± 32.39	68.86-80.29	0.9519	
>40 ≤ 60	284	74.61 ± 33.07	70.74-78.47		
>60	56	73.16 ± 25.74	66.27-80.06.		
**Mean MPR of ADs in MDD patients without HIV/AIDS vs. age group (dispensed items)**					
**Age (years)**	**n**	**Mean ± SD (%)**	**95% Cl**	p**	
>18 ≤ 40	4320	81.39 ± 28.89	80.56-82.28	0.1111	
>40 ≤ 60	10929	80.52 ± 29.85	79.96-81.08		
>60	7582	81.30 ± 29.15	80.65-81.92		

*Two-sample *t*-test.

**One-way ANOVA.

The mean MPR of antidepressants (ADs) was statistical significantly lower *(p* < 0.0001; *d* = 0.2255) in MDD patients with HIV/AIDS (74.43% ± 32.03, 95% Cl: 71.51-77.34) than in patients with only MDD (80.94% ± 29.44, 95% Cl: 80.56-81.33). These differences were of low practical significance. In MDD patients with HIV/AIDS, antidepressant compliance (MPR) was statistical significantly lower with medium practical significance in females (69.35% ± 29.69, 95% Cl: 66.03-72.67) than in males (84.51% ± 34.18, 95% Cl: 79.10-89.91) from the same cohort *(p* < 0.0001; *d* = 0.4755). Age was not a significant predictor of AD compliance in MDD patients with HIV/AIDS (*p* < 0.9519) and those with only MDD (*p =* 0.1111), as shown in Table 2.

In Table 3, a significant association was found between AD class and AD compliance in MDD patients with HIV/AIDS, with compliance figures as follows; tricyclic antidepressants (TCA) 26.83%; selective serotonin re-uptake inhibitors (SSRIs) 44.93%; diverse antidepressants 52.70%; and serotonin and noradrenalin re-uptake inhibitors (SNRIs) 58.57% (*p <* 0.0001; Cramer’s *V* = 0.3450).

**Table 3:** **The influence of AD class on AD compliance in MDD patients with/without HIV/AIDS (dispensed items)**

**MDD patients with HIV/AIDS (dispensed items)**					
**Antidepressant class**	**n**	**Acceptable (%)**	**Unacceptable (%)**	P***	**Cramer’s** ***V***
SSRIs	207	44.93	55.07	<0.0001	0.3450
Diverse antidepressants	148	52.70	37.30		
SNRIs	70	58.57	41.43		
TCA	41	26.83	73.17		
**MDD patients without HIV/AIDS (dispensed items)**					
**Antidepressant class**	**n**	**Acceptable (%)**	**Unacceptable (%)**	P***	**Cramer’s** ***V***
SSRIs	9435	51.64	48.36	0.0001	0.0942
SNRIs	5777	56.36	43.64		
Diverse antidepressants	5460	48.24	51.76		
TCA	2159	43.72	56.28		

***Chi-square.

The following ADs, as depicted in Table 4, were associated with unacceptably low levels of compliance in MDD patients with HIV/AIDS vs. patients with only MDD, as observed from the compliance ratio: amitriptyline 23.53% vs. 39.34% (*p* < 0.0618; Cramer’s *V* = −0.0494); trazodone 27.27% vs. 50.72% (*p* < −0.0239; Cramer’s *V* = −0.0630); paroxetine 28.57% vs. 54.19% (*p* < 0.0197; Cramer’s *V* = −0.0705); duloxetine 36.67% vs. 55.51% (*p* < 0.0391; Cramer’s *V* = −0.0426); escitalopram 44.16% vs. 52.74% (*p* < 0.0.0359; Cramer’s *V* = −0.0254); bupropion 78.26% vs. 50.46% (*p* < 0.0082; Cramer’s *V* = 0.0698) and venlafaxine 75.00% vs. 56.93% (*p* < 0.0217; Cramer’s *V* = 0.0388).

**Table 4:** **Percentage of the top 10 most frequently dispensed antidepressants with acceptable vs. non-acceptable compliance in MDD patients with/without HIV/AIDS (dispensed items)**

	**HIV + MDD**				**MDD**					
	**n**	**Acceptable**	**n**	**Unacceptable**	**n**	**Acceptable**	**n**	**Unacceptable**	P***	**Cramer’s** ***V***
Mirtazapine	40	51.28	38	48.72	848	46.41	979	53.59	0.3987	0.0193
Escitalopram	34	44.16	43	55.84	1781	52.74	1596	47.26	0.1359	−0.0254
Venlafaxine	30	75.00	10	25.00	1972	56.93	1492	43.07	0.0217	0.0388
Citalopram	25	51.02	18	48.92	1002	51.46	945	48.54	0.9511	−0.0014
Fluoxetine	19	41.30	25	58.70	724	47.95	786	52.05	0.3743	−0.0225
Bupropion	18	78.26	5	21.74	714	50.46	701	49.54	0.0082	0.0698
Duloxetine	11	36.67	19	63.33	1284	55.51	1029	44.49	0.0391	−0.0426
Amitriptyline	8	23.53	26	76.47	548	39.34	845	60.66	0.0618	−0.0494
Trazodone	6	27.27	16	72.73	596	50.72	579	49.28	0.0293	−0.0630
Paroxetine	6	28.57	15	71.43	582	54.19	492	45.81	0.0197	−0.0705

***Chi-square.

## Discussion

This study focused on the prevalence of HIV/AIDS-positive patients within the MDD-diagnosed population in this section of the private health sector of South Africa. Secondly, the study investigated how AD compliance is affected by MDD in HIV/AIDS-positive patients when compared to depressed non-HIV/AIDS patients, and whether AD compliance has any correlation with gender and antidepressant class in this population. These goals were attained in that we were able to confirm the co-morbidity between MDD and HIV/AIDS-positive patients; however, due to the strict inclusion criteria, the numbers of patients were small, which provided a limited picture of the overall prevalence between MDD and HIV/AIDS-positive patients.

This study found that patients diagnosed with both HIV/AIDS and MDD (74.43. ± 32.03, 95% Cl: 71.51-77.34) have a statistical significantly lower compliance with AD treatment when compared to patients diagnosed only with MDD (80.94% ± 29.44, 95% Cl: 80.56-81.33). However, these differences is of low practical or clinical significance, meaning that depressed HIV/AIDS patients would have missed approximately two AD doses (~6.5% of a 30-day treatment period) more than the non-HIV/AIDS depressed patient over the same treatment period. The current study confirms that patients suffering from both MDD and HIV/AIDS have a decreased compliance with MDD treatment. This trend is confirmed in the literature as several authors have found similar results, namely that patients suffering from both conditions are less compliant to AD treatment regimens [14,20,55,56]. Our data also seem to suggest, in Table 4, that patients with HIV/AIDS are less compliant with ADs that present with multi-receptor pharmacology, i.e. TCAs (26.83% compliance; Table 3) and paroxetine (compliance 28.57%, Table 4), while they are more compliant with pharmacologically ‘clean’ ADs, such as the venlafaxine (75% compliance, Table 4). The TCAs display a high binding affinity for non-specific receptors such as muscarinic (mAch), histaminic (HA-1) and alpha-1 adrenoceptors, which are responsible for side effects such as dry mouth, constipation and sedation [57]. Similarly, paroxetine demonstrates an affinity for mAch receptors similar to that of imipramine and has marked anticholinergic adverse effects [57]. Our data infer that the reduced compliance with these antidepressants may have a biological basis, in particular an apparent increase in cholinergic sensitivity in the HIV/AIDS population. Indeed, cholinomimetic antibodies of the immunoglobulin (IgA) class are present in HIV/AIDS patients [58,59], suggesting that these patients may indeed be hypersensitive to drugs with activity on the cholinergic system [60]. Importantly, TCA and paroxetine non-compliance with TCAs and paroxetine can evoke a cholinergic overdrive [57,61], which may augment the hyper-cholinergic state present in HIV/AIDS patients, leading to a greater adverse experience and subsequent non-compliance. Furthermore, since MDD is associated with increased cholinergic drive [62,63], it is clear that poor compliance in this population may worsen the mood disorder. For the successful treatment of both MDD and HIV/AIDS, compliance with treatment is therefore of utmost importance. Poor compliance with treatment can lead to a drastic decline in quality of life, increased social impairment, low occupational functioning and heightened social isolation, increased suicide ideation and ultimately to suicide [21,55,56,64-66].

Another interesting result is that the class of antidepressant plays a significant role in predicting compliance when treating patients with MDD and HIV/AIDS. The tricyclic antidepressant (TCA) class is associated with the weakest compliance ratio (26.83%), whereas the SNRIs present with the highest compliance ratio (58.57%). According to our results, SSRIs represent the class of ADs with the highest prescription frequency (44.42%, N = 466). Both escitalopram and citalopram are considered as first-line treatment for treating MDD in HIV/AIDS patients because of a limited effect on the cytochrome P450 system, thereby reducing drug-drug interactions with ARV treatment [67-69]. Moreover, a number of studies have proposed that MDD associated with HIV/AIDS should be treated with fluoxetine and paroxetine [70-72]. However, their use is limited by drug-drug interaction with protease inhibitors (PIs) due to their potent inhibition of CYP2D6, thereby increasing levels of PIs and associated toxicity [57,67,73]. However, in this study, the SSRIs as a group displayed a relatively weak compliance ratio (44.93%; *p* < 0.0001; Table 3) compared to the SNRIs, which displayed a significantly higher compliance ratio (58.57%; *p* < 0.0001; Table 3). These findings indicate that the SSRIs have more adverse effects in this population than originally predicted, possibly related to their anticholinergic (paroxetine), antihistaminic (citalopram) and general serotonergic properties [57]. In fact, considering the latter point, neuropsychiatric symptoms may be evident in HIV-treated patients, especially those on efavirenz, and respond to treatment with the antiserotonergic agent, cyproheptadine, suggesting increased serotonergic activity/sensitivity in these patients [74]. Furthermore, inappropriate AD discontinuation and non-compliance are also associated with increased serotonergic activity [75] that may amplify the adverse experience associated with HIV/AIDS-related neurotransmitter dysregulation. What is more important considering our data is that SNRIs are currently considered a second-line treatment for MDD in HIV/AIDS patients [68,69], while our data (preliminary as they may be) suggest a possible re-evaluation of these guidelines. Indeed, further studies in this regard are warranted.

Only 127 patients (i.e. 0.24% of the total population in the database) met the criteria of co-morbid MDD and HIV/AIDS. Currently, there exists great controversy in the literature regarding the co-morbidity between depression and HIV/AIDS, with a number of articles placing the co-morbidity rate between 0 and 54% [4,6,7,55]. This finding again highlights the complicated co-morbidity between MDD and HIV/AIDS, the different approaches, methodology and reporting of results used in several studies with regard to the selection of a study population, the demographics and the behaviour of the defined population. Furthermore, the low prevalence rate found in this study might suggest a severe under diagnoses of MDD in HIV/AIDS patients and that MDD symptoms might be mistaken rather as symptoms of HIV/AIDS [76,77].

We also observed that female patients (n = 90) were more than double that of male patients (n = 37). Female patients diagnosed with both MDD and HIV/AIDS displayed significantly lower compliance with AD treatment when compared to male patients of the same group, and displayed almost double the prevalence of both MDD and HIV/AIDS than male counterparts. Some of the reasons given for the poor compliance rate to AD treatment in women are the risk of adverse effects (e.g. weight gain and sexual dysfunction), low socio-economic status, younger age, drug abuse and divorce marital status [78]. Similar to our findings, two separate studies found that almost double the number of women with MDD and HIV/AIDS are likely to die from an HIV/AIDS-related cause when compared to HIV-positive women without depression [79], while females have a significantly higher frequency of MDD and HIV/AIDS vs. males with a combined diagnosis [80,81]. Some authors have suggested that lower levels of testosterone might play a role in the large prevalence of MDD in females when compared to men [82]. In support of this, males with hypogonadism have a significantly higher prevalence of MDD and anxiety disorders when compared to healthy males [83,84]. Moreover, testosterone-replacement therapy in hypogonadal men significantly improves these symptoms [83,85,86]. Importantly, studies have found benefits in administering testosterone to depressed women [87,88], thereby supporting the role of testosterone as a causal factor for MDD in women. However, a study in depressed HIV/AIDS patients has shown that testosterone displayed no significant effect on mood [56].

Examining individual ADs dispensed to patients who are suffering from both MDD and HIV/AIDS, mirtazapine was the most frequently dispensed AD (n = 78), followed closely by escitalopram (n = 77). Although mirtazapine is not a first-line AD for the treatment of MDD, its side effect profile seems to play a valuable role in late-stage HIV/AIDS patients with co-morbid MDD, with side effects such as constipation, weight gain and sedation helping to improve diarrhoea, diminished appetite and insomnia, respectively [67].

The strength of the current study is that only patients diagnosed with MDD by a psychiatrist were included in the study population. This article has shed light on the current practice of AD treatment in HIV/AIDS patients with medical aids. The most important findings include that HIV/AIDS with co-morbid MDD have reduced compliance with ADs, especially those exhibiting multi-receptor pharmacology, most notably TCAs and certain SSRIs such as paroxetine. Secondly, MDD patients with co-morbid HIV/AIDS display significantly higher compliance with venlafaxine, contrary to current clinical practice where guidelines suggest SSRIs and SNRIs as first and second line treatment options, respectively. Further clarification with respect to duloxetine is needed as it performed poorly in our analysis. Lastly, female patients are more prone to develop MDD and are notably less compliant with AD treatment.

The authors recognise a few limitations in the current study. Firstly, the numbers of patients with both MDD and HIV/AIDS were relatively small and not a full representation of the national population. Several factors contributed to the small sample size, such as the use of very strict inclusion criteria, only a portion of all the medical aids is listed with the PBM utilised in this study, depressed patients using psychological treatment for MDD were not taken into account, while the study population consisted of only patients contributing to a private medical aid/insurance. Secondly, the use of a database limits the further extraction of patient information regarding the stage of HIV/AIDS in which the patient is and whether there was an improvement or deterioration in the patient’s mood.

## Conclusion

This study confirms that AD compliance is lower in depressed HIV/AIDS vs. depressed non-HIV/AIDS patients, correlating with global trends regarding non-compliance of patients with a chronic disease and co-morbid disorders. However, this study indicated that the clinical and practical significance of the differences is low. Moreover, the fact that the prevalence of MDD patients with co-morbid HIV/AIDS was limited in this study again highlights the need for more research in various population groups with a focus on the socio-economic status of the patients. Although our data are preliminary and warrant further study, it does reveal evidence that venlafaxine may be a valid choice of antidepressant in HIV/AIDS patients suffering from MDD and should be considered as first-line antidepressant in such cases. Moreover, our data suggest that multi-target antidepressants such as TCAs and select SSRIs may increase the risk of non-compliance in this population. Lastly, when prescribing ADs to females, special consideration should be given to counselling them on both their illness and treatment so that they clearly understand the benefits of compliance to treatment.

## Methods

We conducted a prospective, cohort study analysing nationally representative medicine claims data submitted to a privately-owned South African Pharmaceutical Benefit Management (PBM) company. The data represent a third of South African patients with private medical aids. No distinction was made between races. Two groups were distinguished in the database over a six-year study period (1 January 2006 to 31 December 2011), namely patients with only MDD (n = 12 270) and MDD patients with HIV/AIDS (n = 127).

We queried data for patient demographics (gender and date of birth) and pertinent prescription information (such as drug trade name, days supplied, dispensing date, quantity of medicine prescribed, and ICD-10 code per claim). The following automated validation processes were applied by the PBM that ascertained the quality of data: data integrity validation, eligibility management, medicine utilisation and clinical management, pricing and formulary management. There were no missing data fields in the datasets. The variables ‘birth date’ and ‘dispensing date’ were used to calculate the age of patients on the date of treatment and the number of days between refills.

A study population was selected according the following inclusion criteria: patients older than 18 years, MDD should be diagnosed by a psychiatrist supported by an appropriate ICD-10 code. The ICD-10 codes are based on the International Classification of Diseases, 10^th^ edition published by the World Health Organization [89]. In this study, the ICD-10 codes F32 (Depressive episode) and F33 (Recurrent depressive disorder) were used to identify patients with MDD as diagnosed by a psychiatrist, as well as B20-B24 (Human immunodeficiency virus [HIV/AIDS] disease), and all patients on cARV treatment. Thereby it was ensured that data were excluded where ADs may have been used for other indications, such as amitriptyline for the treatment of chronic pain.

For the purpose of this study, the medicine possession ratio (MPR) was used to determine AD compliance of patients. The MPR is a well-established method to calculate drug compliance in pharmacoepidemiological studies, including chronic diseases such as MDD [90], hypertension [91], and schizophrenia [92]. However, it is important to note that the compliance value obtained from the MPR only gives an indication of the possession of medicine by the patient, and that appropriate consumption of medicine is assumed to ensue from possession. The usage of medicine claims data to determine MPR calculations is valuable in that it is acceptably accurate, convenient, objective, non-invasive and relatively inexpensive to obtain when a large study population is needed. It is therefore suitable for the calculation of MPR as an indication of patient compliance with medication therapy [93,94]. However, the MPR has a number of limitations. Firstly, MPR can only be calculated if a patient has filled more than two prescriptions. Secondly, MPR can only assess whether the medication was used consistently. Lastly, the MPR cannot measure whether a patient was compliant with the instructions given by the medical practitioner [95].

The MPR is defined as the number of days for which medication is supplied within the refill interval (medicine treatment period) divided by the number of days in the refill interval [96,97].

$$ \mathrm{M}\mathrm{P}\mathrm{R} = \frac{\mathrm{Number}\ \mathrm{of}\ \mathrm{days}\ \mathrm{in}\ \mathrm{refill}\ \mathrm{in}\mathrm{terval}}{\mathrm{Sum}\ \mathrm{the}\ \mathrm{days}\ \mathrm{of}\ \mathrm{supplied}\ \mathrm{medication}}\times 100 $$

The MPR is considered acceptable if the calculated value is ≥ 80%, but ≤ 110%. An MPR of less than 80% indicates the presence of refill gaps so that possession is considered unacceptably low (undersupply), whereas an MPR greater than 110% is considered unacceptably high (oversupply). Conversely, a patient was considered compliant (acceptable group) with his/her AD treatment if the MPR was ≥ 80% and ≤ 110%, and AD treatment period was longer than 120 days. Therefore, distinguishing between compliant MPR group and patients out of this range will be considered as the non-compliant MPR group or unacceptable group.

Data management and analysis were performed in SAS Version 9.1.3 (SAS Institute, Cary, NC) [98]. All statistical significance was considered with a probability of *p* < 0.05. The practical significance of results was computed when the *p*-value was statistically significant (*p* ≤ 0.05).

Variables (age groups, gender and active ingredients) were expressed using descriptive statistics such as frequencies (n), percentages (%), means, standard deviations and 95% confidence intervals (CI). The patient’s age was determined at time of first dispensing and divided into three groups: > 18 to ≤ 40 years; > 40 to ≤ 60 years; > 60 years.

The dataset is very large, therefore all the statistical methods used in this study rely on the Central Limit Theorem, which states that the average of a large number of independent random variables is approximately normally distributed around the true population mean [99]. Therefore, the two-sample t-test allowed us to compare the mean MPR of male and female patients. The one-way ANOVA was used to test differences between the mean MPR of different age groups and AD classes. It was operationalised with the general linear model (GLM) procedure of the SAS Version 9.1.3. If a difference was indicated, a Tukey’s multiple comparison test was performed to determine which groups differ significantly from each other. Cohen’s d was used to evaluate effect size between means (with d ≥ 0.8 defined as a large effect with practical significance). The Chi-square test (χ2) was used to determine whether an association exists between proportions of two or more groups (compliance vs. active ingredients). The Cramer’s V statistic was used to test the practical significance of this association (with Cramer’s V ≥ 0.5 defined as practical significance).

This study was approved by the Research Ethics Committee of the North-West University (NWU-0046-08-550) and the Board of Directors of the South African Pharmaceutical Benefit Management (PBM). Data were analysed anonymously.
